# Burning Mouth Syndrome: Aetiopathogenesis and Principles of Management

**DOI:** 10.1155/2017/1926269

**Published:** 2017-10-18

**Authors:** L. Feller, J. Fourie, M. Bouckaert, R. A. G. Khammissa, R. Ballyram, J. Lemmer

**Affiliations:** ^1^Department of Periodontology and Oral Medicine, Sefako Makgatho Health Sciences University, Pretoria, South Africa; ^2^Department of Maxillofacial and Oral Surgery, Sefako Makgatho Health Sciences University, Pretoria, South Africa

## Abstract

Burning mouth syndrome (BMS) is a chronic debilitating oral condition characterised by a burning sensation of the oral mucosa in an otherwise apparently normal person. Its aetiology and pathogenesis are obscure, but both psychogenic factors and peripheral and central neuropathies appear to be implicated. There is no cure for BMS, and treatment with either local or systemic medications focuses on the relief of symptoms and on improving quality of life. In recalcitrant cases, psychological/psychiatric intervention may be helpful. In order to improve treatment outcomes, a better understanding of the pathogenesis of this syndrome might provide a basis for the development of more effective management strategies. In this short review, we discuss current knowledge of the diagnosis, aetiopathogenesis, and management of BMS.

## 1. Introduction

The International Association for the Study of Pain (IASP) has described burning mouth syndrome as a chronic condition characterised by a burning sensation of the oral mucosa for which no cause can be found [1]. The anterior part of the tongue is most commonly affected, followed by the labial mucosa and occasionally the palate. The burning pain is often accompanied by tingling or numbness and the sensation of dryness of the mouth [2–4]. Reduced taste intensity and a bitter or a metallic taste are experienced by about two-thirds of those with BMS. Despite all these symptoms, the oral mucosa and the salivary flow rate are normal [5, 6].

The burning sensation of BMS is moderate to severe in intensity, is usually bilaterally symmetrical, and is present every day for most of the day. It is minimal or even absent early in the morning and during mealtimes and it seldom interferes with sleep. In most cases, BMS starts spontaneously [2, 4–7] and continues for several years. Only about 3% of cases resolve over a 5-year observation period and even with treatment only about 30% of affected persons report any improvement [2, 3, 5, 8].

The worldwide prevalence of BMS is unknown because nearly all studies have been of European or North American populations and different diagnostic criteria have been used in different studies [2]. The frequency of BMS increases with age in both women and men and is highest in women aged 60–69 years [9]. The higher frequency of BMS in women (F : M = 5 : 1) [10] may very well be owing to biological, sociocultural, and psychological factors [11].

A diagnosis of BMS can be made only if the oral mucosa is clinically normal and all systemic and local causes for a burning sensation have been excluded (Table 1), bearing in mind that BMS may be superimposed upon a burning sensation of known systemic or local origin [5, 7, 8]. It is essential for treatment purposes thus to differentiate between BMS which is an idiopathic condition and oral mucosal burning sensations secondary to a known cause [12, 13].

**Figure 1:**
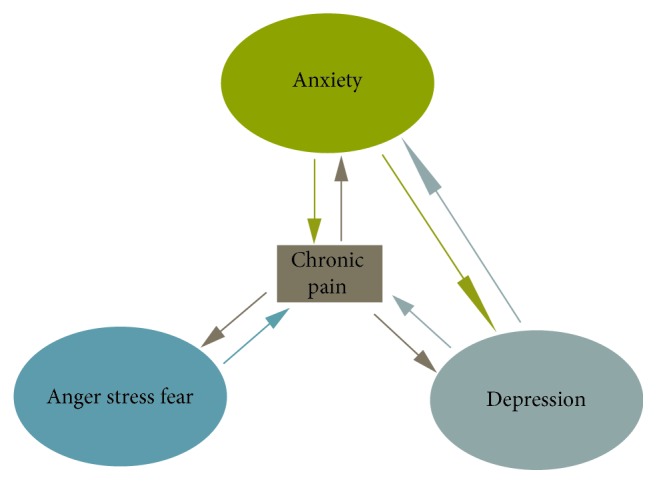
The interrelation between chronic pain, anxiety, depression, and other emotions. The greater the intensity of the pain the greater the suffering, and anxiety, depression, and the stressful emotions may aggravate the experience of pain.

BMS is frequently associated with stressful life events, anxiety, and depressive disorders [10, 14], and as these psychogenic factors can either enhance or reduce perception of pain (Figure 1), BMS can be managed by pharmacological or by psychological means or by a combination of the two [14, 15]. As with other chronic neuropathic pain conditions, BMS can induce or promote psychic symptoms or can itself be a somatic feature of a psychic disorder [11], but it is unclear, however, whether psychogenic factors are primary or secondary in any particular case of BMS.

Systemic and topical medications (Table 2) have been used in the treatment of BMS with varying degrees of success [8]. Psychological/psychiatric intervention should be considered only when BMS does not respond favourably to medication. In particular, cognitive-behavioural therapy which helps the patient to develop pain-coping strategies was found to be beneficial in reducing suffering [5]. Unfortunately, persons with BMS are unwilling to seek psychological treatment because they are convinced that the cause of the burning sensation is in the mouth and is not psychogenic [16].

## 2. Psychological Factors

The chronic burning sensation of BMS has the characteristics of neuropathic pain and is debilitating with a negative impact on quality of life [11]. As attempts of treatment are often unsuccessful, owing to the chronicity of the burning sensation, sufferers tend to consult one doctor after another, but in vain. Consequently some become cancerophobic, believing that either they have or are developing cancer, and this exaggerates their anxiety [5, 17, 18].

With BMS, there are no clinical signs, and as results of laboratory investigations are usually unremarkable, clinicians who are not familiar with BMS often regard the patient as merely emotionally unstable and the complaint is often not taken seriously. This perceived lack of interest only increases the patient's anxiety which in turn increases perception of the pain of BMS [17–19]. Management of BMS should therefore always be empathic and sympathetic with assurance that the condition is not uncommon and is certainly not sinister or life-threatening. Giving basic information about the nature of the complaint and limitations of its treatment and allaying fears, this usually gives the patient a better understanding of the condition and perhaps even some psychogenic relief with consequent reduction in the experience of the pain [14, 17, 19].

It is evident that there is a strong link between BMS and the psychic status. The cause-and-effect relationship between depression, anxiety or neuroticism, and BMS is not clear as the psychic factors may be either causative of or consequential to the oral symptoms [2, 10]. Therefore, in order to achieve best clinical outcomes, treatment should include psychological intervention [20].

One functional magnetic resonance imaging (fMRI) study suggests that in subjects with BMS painful thermal trigeminal stimuli generate different patterns of brain activation in terms of their location and amplitude when compared to healthy control subjects without BMS [21]. Furthermore, another fMRI study investigating the functional connectivity of brain neural circuits associated with pain and emotions in subjects with BMS suggests that these subjects show different patterns of functional brain connectivity in relation to different reported intensities of burning/pain: high intensity burning/pain is associated with increased functional activity of those affective-motivational neural circuits regulating symptoms of depression and anxiety [22].

Some pain modulating neural pathways descending from the cortex, hypothalamus, midbrain, and medulla to the spinal cord, influenced by emotional states such as excitement, stress, anxiety, or depression, can either potentiate or suppress spinal nociceptive pathways and may also have the capacity to spontaneously induce nociceptive signals without peripheral input [23, 24]. This mechanism may be the link between psychogenic factors and BMS pain.

The descending inhibitory pathways of this conditioned pain modulatory system are mediated by serotonin, noradrenalin, gamma aminobutyric acid (GABA), and enkephalins. Thus, both a nonpharmacological approach (e.g., cognitive-behavioural therapy and mindfulness meditation) and pharmacological agents targeting neuronal central sensitization pathways (e.g., anticonvulsants, serotonin noradrenalin reuptake inhibitors (SNRIs), GABA receptor agonists, and N-methyl-D-aspartate (NMDA) receptor antagonists), alone or in combination, are beneficial in the treatment of neuropathic pain conditions, including BMS [15].

Persistent pain which does not significantly respond to treatment, such as in the case of BMS, is debilitating in that it negatively affects the psyche, evoking feelings of vulnerability, helplessness, and desperation partly because of the fear that the pain will never let up or might even become worse. Such negative emotions can overwhelm the BMS sufferer causing anxiety and depression, interfering with everyday activities, and significantly reducing the quality of life [19]. BMS pain is associated with irritability, fatigue, anorexia, diminished social activity, and depression. Psychological/psychiatric intervention might therefore be beneficial [4, 8]. Furthermore, the strong association between anxiety and depression on the one hand and BMS pain on the other hand provides the biological rationale for the use of anxiolytics and antidepressants in the treatment of BMS [11].

Additional research is necessary into the roles of genetic factors, familial patterns, and the nature of the complex interactions between stressful life events, personality, comorbid psychic factors, and the lack of social support in the pathogenesis of BMS as it may allow for the identification of predictors of successful treatment [10, 25].

## 3. Pathogenesis

In the pathogenesis of BMS, apart from psychogenic factors, both peripheral and central neuropathies appear to play a role, but the balance between central and peripheral neuropathies varies from case to case [27]. It is probable that genetic and environmental factors play an important role in determining individual differences in the experience of pain [15].

In persons with BMS who are anxious or depressed and who do not have immediate relief after local anaesthetic regional nerve block, or after topical treatment with capsaicin or with clonazepam, central neuropathy is probably the dominant mechanism of the pain [27]. As in most persons with BMS, local treatment for the burning sensation is only transiently successful; central neuropathy, to different degrees, more often than not plays a significant role in BMS [4, 28].

Several neuronal dysfunctions are linked to BMS pain. The lingual mucosa exhibits a decreased number of small-diameter nerve fibres; the remaining small-diameter nerve fibres show upregulation of the transient receptor potential subfamily member V 1 (TRPV1) ion channel, and upregulation of the P2X3 receptors and of nerve growth factor (NGF). These explain the role of trigeminal small-fibre sensory neuropathy in the pathogenesis of BMS [4, 8, 9, 27, 29].

TRPV1 channels are mostly found in nociceptive terminals of peripheral A*δ* and C fibres but also centrally in the dorsal root and trigeminal ganglia. They respond to chemical irritants including the chilli pepper ingredient capsaicin [30]. P2X3 ion channel receptors are expressed by a subpopulation of small-diameter primary nociceptors in the trigeminal nervous system and when activated by adenosine triphosphate (ATP) they can evoke a sensation of burning pain [31, 32].

It has been further proposed that downregulation of central dopaminergic pain-inhibitory pathways also plays a role in the pathogenesis of BMS [2, 4, 27], particularly in persons with anxiety or depression, which are both associated with dysregulation of central mood-mediating dopaminergic pathways.

In the context of persistent peripheral neuropathy, the central afferent nociceptor terminals in the dorsal horn of the spinal cord release excitatory biological mediators which can activate postsynaptic NMDA receptors which under physiological conditions are silent, thus resulting in central sensitization with increased excitability [23]. There may also be a decrease in the functional activity of the GABA-mediated pain-inhibitory interneuron circuits in the dorsal horn of the spinal cord which under physiological circumstances inhibit the glutamate/NMDA-mediated central sensitization [23], possibly contributing to the neuropathic pain of BMS [5].

Thus, central sensitization characterised by structural and functional neural plasticity results in increased excitability and increased tonic activity of central nociceptive neurons, playing an important role in the pathogenesis of BMS [2, 14]. However, surprisingly, despite the possible roles of central sensitization and of psychogenic factors such as anxiety or depression in BMS neuropathic pain, it appears that, in persons with BMS, the co-occurrence of other chronic neuropathic pain disorders (central sensitivity syndromes) including fibromyalgia, atypical facial pain, trigeminal neuralgia, temporomandibular joint pain, back pain, and vulvodynia is rare [15, 33]. This suggests that the neural pathogenic mechanisms of BMS are distinct, probably localised somewhere in the trigeminal nerve pathway [33].

## 4. Treatment

Patients should be told that BMS is a complex disorder for which there is no cure and treatment is purely symptomatic, and therefore expectations of the outcome of treatment should not be unrealistic [28]. As the aetiopathogenesis of BMS is poorly understood, there is no standard treatment, and treatment is empirical and largely based on personal and expert opinion [14].

### 4.1. Topical and Systemic Medication

Capsaicin is the “hot” component of chilli pepper and has been used both topically and systemically in the treatment of BMS, reportedly to bring about symptomatic relief. Topical capsaicin has the capacity to bind to the TRPV1 ion channels of small-diameter peripheral sensory nerve fibres, mediating desensitization of afferent nociceptors, and causing reversible degeneration of peripheral sensory nerve endings, with consequent reduction in the syndromal burning pain sensation [28, 30]. It has also been reported that capsaicin can downregulate the biosynthesis of neurotransmitters and their axonal transport by primary nociceptors, thus inhibiting central sensitization in response to peripheral nociceptive stimuli [30, 34]. Systemic capsaicin is not well tolerated because of its side effects and therefore is seldom used.

Clonazepam, a benzodiazepine, is a GABAnergic agonist, which activates pain-inhibitory pathways in the spinal cord and in peripheral nociceptors. When applied topically to the oral mucosa, clonazepam is thought to decrease excitability of peripheral sensory nerve fibres, and when given systemically, it has central sedative, anxiolytic, and analgesic effects. The use of both topical and systemic clonazepam has been reported to reduce the intensity of the pain of BMS [4, 5, 8, 26, 35, 36].

Antidepressants are often used to treat BMS most probably because of their documented effects in reducing the intensity of neuropathic pain and because of the close association between BMS and generalized anxiety and depressive disorders [3]. Studies have shown that tricyclic antidepressants (e.g., amitriptyline) bring relief to a significant number of sufferers of BMS [4, 26].

Alpha lipoic acid, a potent antioxidant, has been shown to be beneficial in the treatment of BMS, either alone or particularly in combination with anticonvulsants or with psychotherapy (cognitive behaviour therapy) [28]. The anticonvulsant gabapentin which is often used for the treatment of neuropathic pain disorders has been shown also to reduce BMS pain [8].

### 4.2. Psychologic/Psychiatric Intervention

Individual or small-group cognitive-behavioural therapy has been shown to reduce the intensity of the pain of the BMS in a significant number of sufferers, either alone or with medications [4, 37].

**Figure 2:**
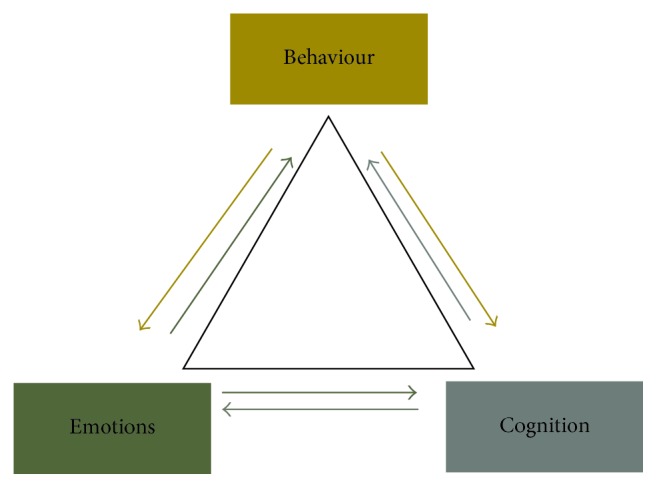
The interrelationship between emotion, behaviour, and cognition.

Cognitive-behavioural therapy is a directed, structured, short-term psychological treatment aimed at correcting dysfunctional emotional responses such as pain, fear, helplessness, vulnerability, or exhaustion, by changing thoughts and behaviours [25]. The rationale for this therapy is based on the concept that cognition, emotion, and behaviour are interrelated, forming a “complex adaptive system” (Figure 2). Accordingly, modifications in dysfunctional cognition and/or behaviour may be beneficial in correcting irrational emotional responses, and modifying cognition may correct dysfunctional behaviour [25].

With cognitive-behavioural therapy, harmful thoughts and problematic behaviours are identified, and the dysfunctional relationship between cause and disordered emotional response is explained to the patient. An understanding of the mechanisms that drive dysregulated relationships between thought, behaviour, and emotion may decrease the level of anxiety. Cognitive-behavioural therapy can undoubtedly be an effective part of the management of BMS [2, 25, 37].

As it is evident that sometimes psychogenic factors are closely if not causally associated with the neuropathic pain of BMS, and if antidepressant, anxiolytic, or anticonvulsant agents are indicated, then management should be by a psychiatrist.

## 5. Conclusion

The pathogenesis of BMS is complex involving psychogenic factors and dysregulated peripheral and central pain pathways. Genetic factors determining the function of neural pain pathways may play an important role in individual susceptibility to BMS, and while there is no standard treatment protocol for its management, both drugs and psychological services may be required.

## Figures and Tables

**Table 1:** Some systemic and local causes of a burning sensation in the mouth, which, therefore, by definition is not BMS [4, 8, 12, 13, 18, 26].

(1) Oral mucosal conditions
(i) Erythema/erosion of whatever cause
(ii) Atrophic tongue
(iii) Candidosis
(iv) Geographic tongue
(v) Lichen planus
(vi) Pemphigoid, pemphigus
(2) Parafunctional habits
(i) Cheek sucking
(ii) Tongue thrusting
(3) Trauma: mechanical, chemical, thermal
(4) Xerostomia and altered salivary quality
(i) Radiotherapy
(ii) Chemotherapy
(iii) Other drugs
(iv) Sjögren's syndrome
(5) Systemic factors
(i) Diabetes
(ii) Decreased levels of vitamins B1, B2, B12, folate, iron, zinc
(iii) Abnormal thyroid function
(iv) Allergic reaction to food or dental materials
(v) Lichenoid tissue reactions
(vi) Autoimmune conditions
(vii) Hormonal disturbances
(viii) Parkinson disease
(6) Drugs
(i) Paroxetine
(ii) Angiotensin-converting enzyme inhibitors
(7) Local nerve damage
(i) Chemotherapy-associated neuropathy
(ii) Local physical irritation
(8) Various peripheral or central neuropathies

**Table 2:** Available agents or strategies for the management of BMS based on expert opinion and common clinical practice. Adapted from [2].

*Pharmacological agents*

(1) *Topical*
(i) Clonazepam
(ii) Capsaicin
(iii) Doxepin
(iv) Lidocaine
(2) *Systemic*
(i) Tricyclic antidepressants
(ii) Selective serotonin reuptake inhibitors
(iii) Serotonin-adrenalin reuptake inhibitors
(iv) Anticonvulsants (e.g., gabapentin)
(v) Opioids
(vi) Benzodiazepines
(vii) Alpha-lipoic acid

*Nonpharmacological therapy*

(1) Cognitive-behavioural therapy
(2) Mindfulness meditation
(3) Other relaxation techniques
